# In Situ Investigation of a Self-Accelerated Cocrystal Formation by Grinding Pyrazinamide with Oxalic Acid

**DOI:** 10.3390/molecules21070917

**Published:** 2016-07-14

**Authors:** Hannes Kulla, Sebastian Greiser, Sigrid Benemann, Klaus Rademann, Franziska Emmerling

**Affiliations:** 1 Federal Institute for Materials Research and Testing (BAM), Richard-Willstaetter-Str. 11, 12489 Berlin, Germany; hannes.kulla@bam.de (H.K.); sebastian.greiser@bam.de (S.G.); sigrid.benemann@bam.de (S.B.); 2 Department of Chemistry, Humboldt-Universität zu Berlin, Brook-Taylor-Str. 2, 12489 Berlin, Germany; klaus.rademann@hu-berlin.de

**Keywords:** in situ, cocrystal, mechanochemistry, hydrate, pyrazinamide

## Abstract

A new cocrystal of pyrazinamide with oxalic acid was prepared mechanochemically and characterized by PXRD, Raman spectroscopy, solid-state NMR spectroscopy, DTA-TG, and SEM. Based on powder X-ray diffraction data the structure was solved. The formation pathway of the reaction was studied in situ using combined synchrotron PXRD and Raman spectroscopy. Using oxalic acid dihydrate the initially neat grinding turned into a rapid self-accelerated liquid-assisted grinding process by the release of crystallization water. Under these conditions, the cocrystal was formed directly within two minutes.

## 1. Introduction

Mechanochemistry—the reaction of solid state components by grinding—has evolved to an important method for the synthesis and screening of new crystalline materials. The growing popularity is strongly related to the increasing interest in the research of cocrystals, especially of active pharmaceutical ingredients (API). According to the FDA, cocrystals are crystalline materials composed of two or more molecules in the same crystal lattice [1]. In contrast to salts, components of cocrystals are in a neutral state stabilized by nonionic interactions such as hydrogen bonds, π-π-stacking, or halogen bonds [2,3]. Cocrystallization of an API with a so-called coformer, an ideally non-toxic second molecule offers a great potential for improving physiochemical properties like solubility, thermal stability, and bioavailability without altering the therapeutic effect of the API. The occurrence of polymorphism describing compounds having the same chemical composition but different crystal structures even broadens the diversity of new crystalline materials. It was described previously that the polymorphic outcome can be controlled by grinding with solvents of different polarity [4,5]. This liquid-assisted grinding (LAG) approach enables access to different polymorphs and may also promote reactions which were unsuccessful under neat grinding [6]. Moreover, LAG is preferred to neat grinding as products are often formed faster and more crystalline [7,8]. Solvated reactants such as hydrates could possibly play the same role [6,9,10,11,12,13].

Mechanochemistry presents an effective method for the screening of cocrystals and their polymorphs [14,15] facilitating products that may not be obtained from solution or melt [6,7,16,17,18]. The formation pathways of mechanochemistry are still not completely understood. First ex situ studies suggested a stepwise formation process [14,19,20]. A drawback of these experiments is the interruption of the reaction’s progress leading potentially to different products than a continuous reaction [21]. Recently, an approach was introduced combining in situ powder X-ray diffraction (PXRD) and Raman spectroscopy to study milling syntheses. In general, these investigations support a stepwise formation [4,22,23,24,25]. For other cocrystal systems a rapid conversion after a long induction period was observed [26]. Pyrazinamide is one of the most important drugs for treatment of tuberculosis. Cocrystals of the API pyrazinamide (PZA) are widely studied documented by 25 entries in the Cambridge Structure Database (CSD, version 5.37, November 2015). Pyrazinamide forms cocrystals with aromatic acids [27,28,29] and dicarboxylic acids which increase the solubility and dissolution rate of PZA [30,31]. In this study, we present the in situ investigation of grinding pyrazinamide with oxalic acid. A new cocrystal in a molar ratio of 1:1 was obtained. Based on high-resolution PXRD the crystal structure could be solved showing rare case of acid-acid homosynthons. The solid form was thoroughly characterized by Raman spectroscopy, solid-state NMR (ssNMR) spectroscopy, differential thermal analysis (DTA) with coupled thermogravimetric (TG) analysis, and scanning electron microscopy (SEM). In situ investigations using PXRD coupled with Raman spectroscopy were conducted to elucidate the formation pathways of a pyrazinamide cocrystal for the first time. The reaction progress of neat grinding PZA with oxalic acid dihydrate is compared to that of oxalic acid anhydrous. For oxalic acid dihydrate, the in situ data indicates a direct self-initiated liquid-assisted grinding process.

## 2. Results and Discussion

### 2.1. Characterization of the PZA:OA (1:1) Cocrystal

**Figure 1:**
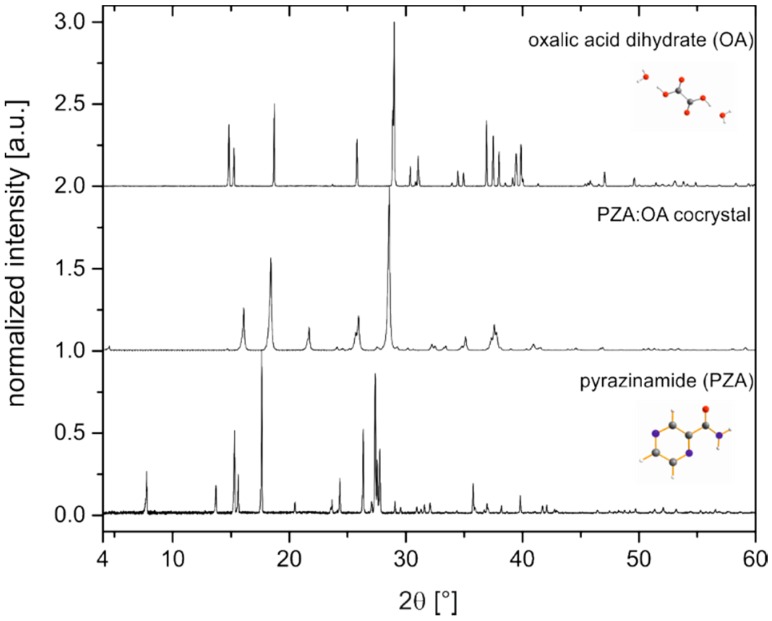
Powder patterns of the PZA:OA (1:1) cocrystal (**center**) and the reactants pyrazinamide (**bottom**) and oxalic acid dihydrate (**top**).

Cocrystallization of pyrazinamide (PZA) with oxalic acid (OA) was conducted by neat and liquid-assisted grinding (LAG). Various stoichiometries (1:1, 2:1, 1:2 and 4:1) and solvents of different polarity (acetone, acetonitrile, chloroform, dioxane, ethanol, and water) were tested to also identify possible polymorphs. This screening resulted in a new cocrystal of pyrazinamide:oxalic acid 1:1. The crystallite sizes and morphology of the final product are reflected in the SEM images (Figure S1). Previous attempts to obtain this cocrystal from solution failed due to precipitation of PZA [30]. When the solubility is low or reactants are not miscible, grinding is superior to solution methods. The powder X-ray diffraction pattern (PXRD) of the new compound in comparison to the PXRD patterns of the reactants, PZA and oxalic acid dihydrate, is depicted in Figure 1.

**Figure 2:**
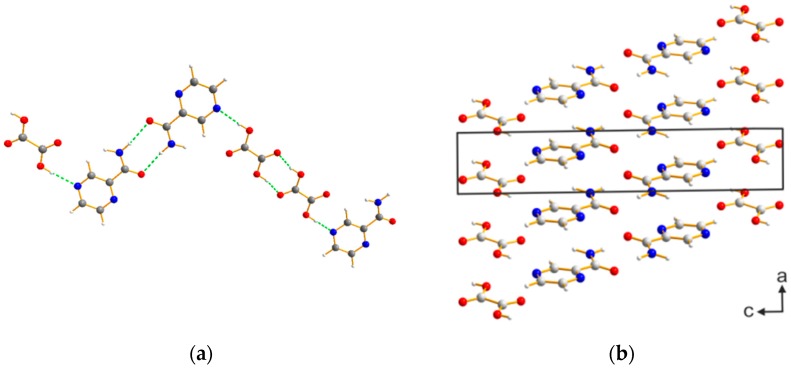
(**a**) Hydrogen bond interactions indicated by green dashed lines (**b**) structure of the PZA:OA (1:1) cocrystal, view along the b-axis.

No reflections of the reactants can be observed in the powder pattern of the cocrystal, indicating the completeness of the reaction. The cocrystal structure was successfully determined from the powder pattern followed by a Rietveld refinement. The resulting crystal structure is presented in Figure 2.

The corresponding Rietveld refinement indicates a good agreement between the simulated and the measured powder patterns (Figure S2). The PZA:OA cocrystal crystallizes in the triclinic spacegroup P1 with Z = 2, (a = 3.6848(3) Å, b = 6.0879(9) Å, c = 19.646(3) Å, α = 80.708(5), β = 90.652(6), γ = 88.793(6), V = 434.784 Å3. The asymmetric unit (ASU) consists of a molecule of PZA and OA. One molecule of PZA forms a dimeric unit by R_2_^2^(8) supramolecular homosynthon through hydrogen bond interactions with the amide-group (N-H···O, d_D···A_ = 2.242 Å). This dimeric unit is then connected to two OA molecules by means of O-H···N (d_D···A_ = 2.098 Å) hydrogen bond interactions. The OA molecules form a homosynthon with a second molecule of OA via OH···O (d_D···A_ = 1.698 Å) hydrogen bond interactions. The dimers are also connected to adjacent PZA molecules by means of an O-H···N (d_D···A_ = 2.098 Å) hydrogen bonds. The resulting structure consists of parallel zig-zag chains stabilized by π-π stacking running along the (101).

**Figure 3:**
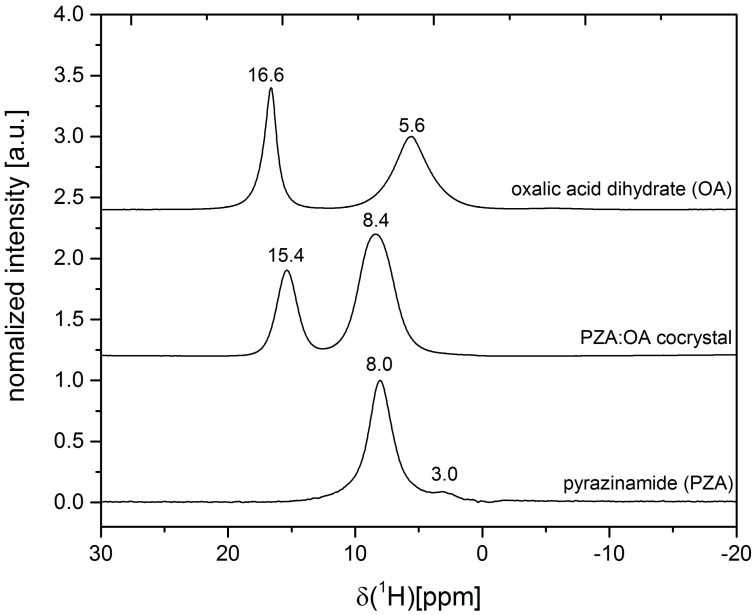
Solid-state NMR spectra of the PZA:OA (1:1) cocrystal (**center**) and the reactants pyrazinamide (**bottom**) and oxalic acid dihydrate (**top**).

The absence of water in the crystal structure is evident from DTA-TG measurements (Figure S3). The first endothermic signal in the DTA of the PZA:OA cocrystal at 217 °C is accompanied by a complete loss of mass, suggesting decomposition of the sample. Apparently, the OA molecules are stabilized in the cocrystal as the temperature is 18 °C above the decomposition temperature of pure oxalic acid (Figure S4). This effect can be related to additional hydrogen bond interactions in the cocrystal resulting in a line broadening in the ^1^H ssNMR spectrum of the PZA:OA cocrystal (Figure 3). The ssNMR measurements also confirm the absence of water in the crystal structure. The water signal at 5.6 ppm present in the spectrum of oxalic acid dihydrate is missing in the cocrystal. Based on the ssNMR spectrum, a salt formation can be excluded. The proton signal of OA at 16.6 ppm is shifted in the cocrystal to 15.4 ppm suggesting a reduced electron density at the carboxylic acid group.

In the Raman spectra of the cocrystal, only the band referring to the carboxylate deformation vibration of oxalic acid dihydrate at 478 cm^−1^ [32] is noticeably shifted to 458 cm**^−^**^1^ compared to the spectra of the reactants (Figure S5). Protonation of pyrazinamide in the cocrystal is therefore unlikely.

### 2.2. In Situ Investigation of the PZA:OA (1:1) Cocrystal

**Figure 4:**
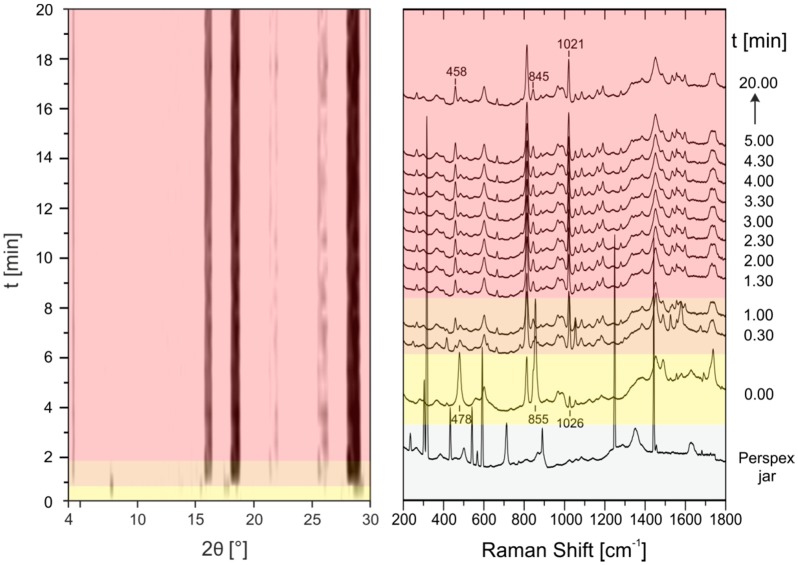
Time resolved investigation of the synthesis process of the PZA:OA (1:1) cocrystal obtained by neat grinding of PZA with oxalic acid dihydrate followed in situ by synchrotron XRD (**left**) and Raman spectroscopy (**right**). The Raman spectrum of the empty Perspex jar (gray) indicates which modes of the following synthesis process arise from the sample holder and which from the reaction mixture. Yellow: reactants; orange: reactants and product; red: product.

For in situ investigations of the cocrystallization the steel jar was replaced by a Perspex jar. Perspex fulfills two aspects for the in situ measurements (i) transparency for X-rays and Raman laser radiation and (ii) sufficient resistance to the chemical and mechanical impact. Under these conditions, simultaneous in situ measurements of X-ray powder patterns using synchrotron radiation and Raman spectra every 30 s during grinding are possible (see Figure S6 for a scheme of the setup). This combination allows the evaluation of mechanochemical formation pathways. In a typical experiment, 1 g of the reaction mixture was ground in a ball mill (Pulverisette 23, Fritsch, Germany) in a Perspex jar for 20 min at 50 Hz. In the case of neat grinding an equimolar mixture of pyrazinamide with oxalic acid dihydrate cocrystal formation occurs very rapidly (Figure 4), (see Figure S7 for an enhanced image of the PXRD patterns between 0 and 5 min).

**Figure 5:**
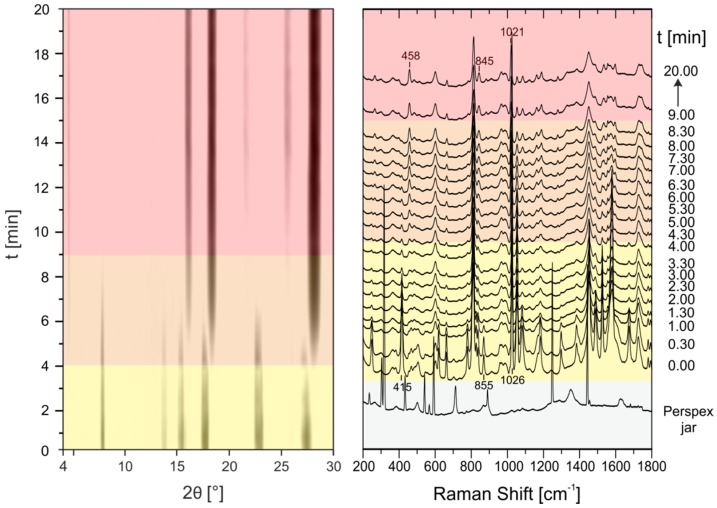
Time resolved investigation of the synthesis process of the PZA:OA (1:1) cocrystal obtained by neat grinding of PZA with oxalic acid anhydrous followed in situ by synchrotron XRD (**left**) and Raman spectroscopy (**right**). The Raman spectrum of the empty Perspex jar (gray) indicates which modes of the following synthesis process arise from the sample holder and which from the reaction mixture. Yellow: reactants; orange: reactants and product; red: product.

After a short induction phase with only reactants present (Figure 4, left, yellow), new reflections that can be assigned to the formation of the PZA:OA cocrystal already appear after 30 s (orange). After another minute of grinding, the reaction is completed (red). The Raman measurements are in good agreement to X-ray powder data. The band of the carboxylate deformation vibration of oxalic acid dihydrate at 478 cm**^−^**^1^ shifts to 458 cm**^−^**^1^ within the first 30 s which is characteristic for the cocrystal formation. Bands referring to the vibration modes of pyrazinamide at 855 cm**^−^**^1^ and 1026 cm**^−^**^1^ are only shifted slightly suggesting cocrystal formation. Based on the time resolved diffraction powder patterns and the Raman spectra, a direct reaction without formation of any intermediates can be deduced. When the reaction is performed under neat conditions with oxalic acid anhydrous instead of the dihydrate the reaction kinetics are remarkably slowed down (Figure 5).

In this case, the cocrystal is only formed after 4 min (orange) and the reaction takes 9 min in total to be finished (red). In the Raman spectra conversion of pyrazinamide can be followed by the disappearing band of PZA at 415 cm**^−^**^1^. Apparently, crystallization water in oxalic acid induces a liquid-assisted grinding process that enhances the conversion rate of the reactants. This behavior has been previously observed by the Boldyreva group [12]. Only recently, the group of Halasz confirmed this reaction mechanism of a solvated reactant for the formation of a coordination polymer by in situ PXRD [13]. However, to the best of our knowledge, the herein provided in situ measurements are the first experimental evidence for an initially neat grinding cocrystal reaction that is transformed into a liquid-assisted grinding process by the release of crystallization water.

## 3. Materials and Methods

### 3.1. Materials

Pyrazinamide, C_5_H_5_N_3_O, (Merck, Darmstadt, Germany), oxalic acid anhydrous, C_2_H_2_O_4_, (98%, Acros Organics, Geel, Belgium), oxalic acid dihydrate, C_2_H_2_O_4_·2 H_2_O, (≥99+%, Acros Organics, Geel, Belgium) were purchased commercially and used without further purification.

### 3.2. Methods

#### 3.2.1. Mechanochemical Synthesis

The synthesis was conducted by neat and liquid-assisted grinding (LAG) in a ball mill (Pulverisette 23, Fritsch, Germany) at a frequency of 50 Hz for 20 min. Starting materials with a total load of 1 g were weighed in a molar ratio of 1:1 into a 10 mL steel vessel together with two 10 mm steel balls.

#### 3.2.2. PXRD Measurements

Final products were investigated by PXRD after drying. In principle measurements were carried out on a D8 diffractometer (Bruker AXS, Karlsruhe, Germany) in transmission geometry in a 2Ɵ range from 5 to 60°, with a step size of 0.009°, using Cu K_α1_ (λ = 1.54056 Å) radiation. For a better Rietveld refinement of the pyrazinamide:oxalic acid cocrystal a PXRD measurement was also performed on a StadiMP diffractometer (STOE, Darmstadt, Germany), equipped with a Ge(111) crystal monochromator and a Mythen 1K (Dectris) detector, in transmission geometry using Cu K_α1_ (λ = 1.54056 Å) radiation. The crystal structure was solved based on the PXRD pattern using the simulated annealing routine implemented in DASH [33]. For Indexing and Rietveld refinement the TOPAS software was applied [34]. CCDC 1486471 contains the supplementary crystallographic data for the cocrystal pyrazinamide:oxalic acid 1:1. These data can be obtained free of charge via http://www.ccdc.cam.ac.uk/conts/retrieving.html (or from the CCDC, 12 Union Road, Cambridge CB2 1EZ, UK; Fax: +44-1223-336033; E-mail: deposit@ccdc.cam.ac.uk).

#### 3.2.3. In Situ Synchrotron XRD

The in situ X-ray diffraction experiments were performed at the µSpot beamline (BESSY II, Helmholtz Centre Berlin for Materials and Energy) in a ball mill (Pulverisette 23, Fritsch, Germany) in a Perspex jar for 20 min at 50 Hz. For the experiments a beam diameter of 100 μm at a photon flux of 1 × 109 s**^−^**^1^ at a ring current of 100 mA was used. The experiments were performed with a wavelength of 1.000 Å using a double crystal monochromator (Si 111). The spot size on the sample was 200 µm. Scattered intensities were collected with a two-dimensional X-ray detector (MarMosaic, CCD 3072 × 3072 pixels, pixel size 73 µm) [35]. Measurements were carried out every 30 s with a delay time of three or four seconds between two measurements. For clarity, the reaction times were given as full 30 s in the text of the results. The obtained scattering images were processed employing an algorithm of the computer program FIT2D [36]. For the graphical representations, q values were transformed to the diffraction angle 2θ (Cu K_α1_) to provide a direct comparison to results obtained by XRD experiments performed with Cu radiation in the laboratory. The resulting patterns (2theta angle vs. intensity) were analyzed and plotted using the evaluation software EVA [37]. The XRD plots are background corrected.

#### 3.2.4. Raman Spectroscopy

Raman measurements were performed using a Raman RXN1™ Analyser (Kaiser Optical Systems, Lyon, France). The spectra were collected using a laser with a wavelength of λ = 785 nm and a contactless probe head (working distance 1.5 cm, spot size 1.0 mm). Raman spectra were recorded with an acquisition time of 5 s and five accumulations. NIR excitation radiation at λ = 785 nm and an irradiation of 6.6 W/cm^2^ were performed.

#### 3.2.5. ssNMR Spectroscopy

^1^H-NMR measurements were conducted on a Bruker AVANCE 600 spectrometer (Bruker Corporation, Billerica, MA, USA) using a 2.5 mm double-bearing magic angle spinning (MAS) probe (Bruker Biospin) and applying a spinning speed of 25 kHz. The spectra were recorded with a π/2 pulse length of 2.75 µs, a recycle delay of 300 s and an accumulation number of 8. An empty rotor was measured to suppress and probe the background. Adamantane was used as a secondary field standard with a chemical shift of 1.78 ppm.

#### 3.2.6. SEM

SEM images were obtained using a scanning electron microscope ZEISS SUPRA 40 (Carl Zeiss AG, Oberkochen, Germany) equipped with a thermal field emission cathode (Schottky-emitter, ZrO/W-cathode). The acceleration voltage was set to 10 kV and the working distance was between 6.0 mm and 6.1 mm. The images were adapted with an in-lens secondary electron detector, a SE2 secondary electron detector and a QBSD back-scatter detector. In addition, the scanning electron microscope is equipped with the energy dispersive X-ray spectrometers Thermo NSS (SiLi 5665) and Bruker X-Flash 5010 3403, Quantax 400 (Bruker Corporation, Billerica, MA, USA).

#### 3.2.7. DTA-TG Analysis

DTA and TGA measurements were conducted using a thermobalance SETARAM TAG24 (SETARAM, Caluire, France) in 1600 °C equipment. The measurements were performed in an open Pt crucible under Ar/synthetic air flow with a heating rate of 10 °C min^−1^ and a cooling rate of 30 °C min**^−^**^1^. Subsequently, a second heating and cooling cycle under the same conditions were performed. The measurement of the second cycle was subtracted for correction of buoyancy effect.

## 4. Conclusions

A new cocrystal of pyrazinamide with oxalic acid in a stoichiometry of 1:1 was synthesized by ball milling. The crystal structure was solved from powder diffraction data. The final product was thoroughly analyzed by PXRD, Raman spectroscopy, ssNMR, DTA-TG, and SEM. Furthermore, in situ investigations using the combination of time resolved PXRD and Raman spectroscopy were performed to elucidate the mechanochemical reaction pathways. For the cocrystal of pyrazinamide:oxalic acid, a direct reaction from the reactants to the cocrystal is observed. A remarkable increase in reaction kinetics for the hydrate is assessed, comparing the neat grinding process with oxalic acid anhydrous versus oxalic acid dihydrate as the coformer. This can be related to the release of water from the hydrate transforming the initially neat grinding into a rapid self-accelerated liquid-assisted grinding process. Based on real time in situ measurements this behavior of a solvated species could be confirmed for the first time for a cocrystal reaction. When available hydrates may be used as a safe and direct source for liquid-assisted grinding. Mechanochemistry presents a fast and effective way to obtain new solid state forms that may not be obtained by solution methods for reasons of solubility or immiscibility of reactants. In situ investigations combining PXRD and Raman spectroscopy provide a deeper understanding of mechanochemical formation pathways.

## Supplementary Materials

Supplementary materials can be accessed at: http://www.mdpi.com/1420-3049/21/7/917/s1.

## Abbreviations

The following abbreviations are used in this manuscript:

API	active pharmaceutical ingredient
CSD	Cambridge Structure Database
DTA-TG	differential thermal analysis with coupled thermogravimetric analysis
FDA	U.S. Food and Drug administration
LAG	liquid-assisted grinding
MAS	magic angle spinning
NIR	near infrared
OA	oxalic acid
PXRD	powder X-ray diffraction
PZA	pyrazinamide
SEM	scanning electron microscopy
ssNMR	solid-state NMR spectroscopy
