# Supplementary material for: In Situ Investigation of a Self-Accelerated Cocrystal Formation by Grinding Pyrazinamide with Oxalic Acid
Source: Molecules. 2016 Jul 14;21(7):917. doi: 10.3390/molecules21070917 (PMC6274108; doi:10.3390/molecules21070917)
Supplement: Supplementary file 1 [file molecules-21-00917-s001.pdf]

Supplementary Materials: In Situ Investigation of a Self-Accelerated Cocrystal Formation by Grinding Pyrazinamide with Oxalic Acid

Hannes Kulla, Sebastian Greiser, Sigrid Benemann, Klaus Rademann and Franziska Emmerling

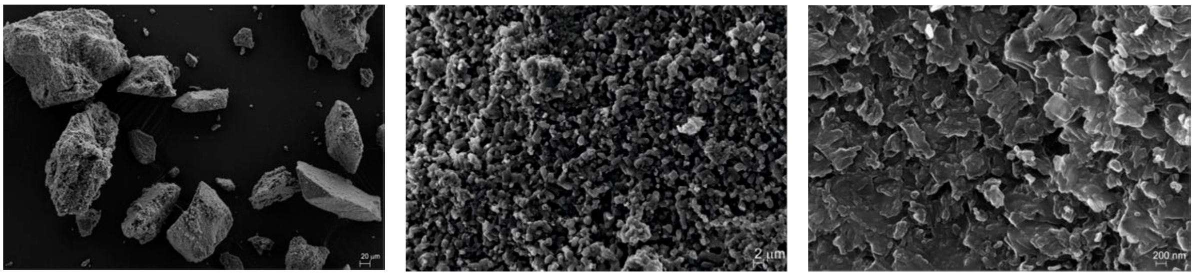

(**a**) (**b**) (**c**)

Figure S1. SEM images of the PZA:OA (1:1) cocrystal at different magnifications (**a**) scalebar 20 μm (**b**) 2 μm and (**c**) 200 nm.

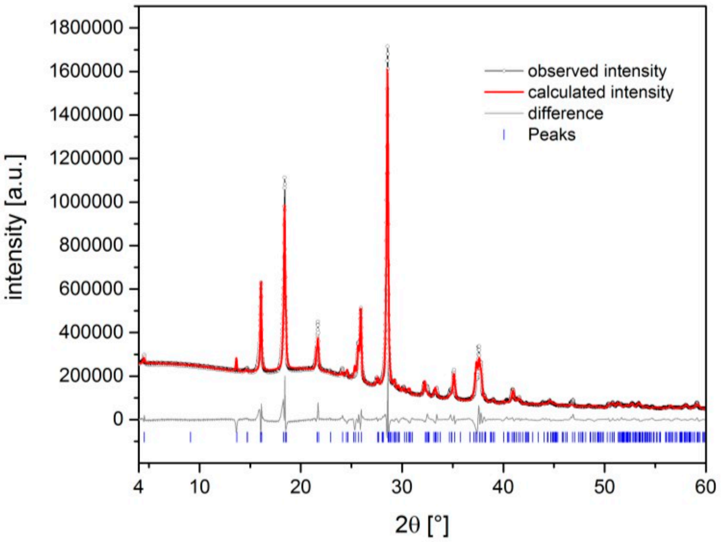

Figure S2. Rietveld refinement of the crystal structure of the PZA:OA cocrystal. Scattered X-ray intensity for the PZA:OA (1:1) cocrystal under ambient conditions as a function of the diffraction angle 2θ. The observed pattern (black circles), the best Rietveld fit profile (red line), the reflection positions (blue tick marks), and the difference curve (grey line) between the observed and calculated profiles are depicted. The wavelength was λ = 1.54056 Å (Cu $K_{\alpha 1}$). The R-values are R_p = 3.2% and R_{wp} = 5.3%; R_p, and R_{wp} refer to the Rietveld criteria of fit for profile and weighted profile defined by Langford and Louer [1].

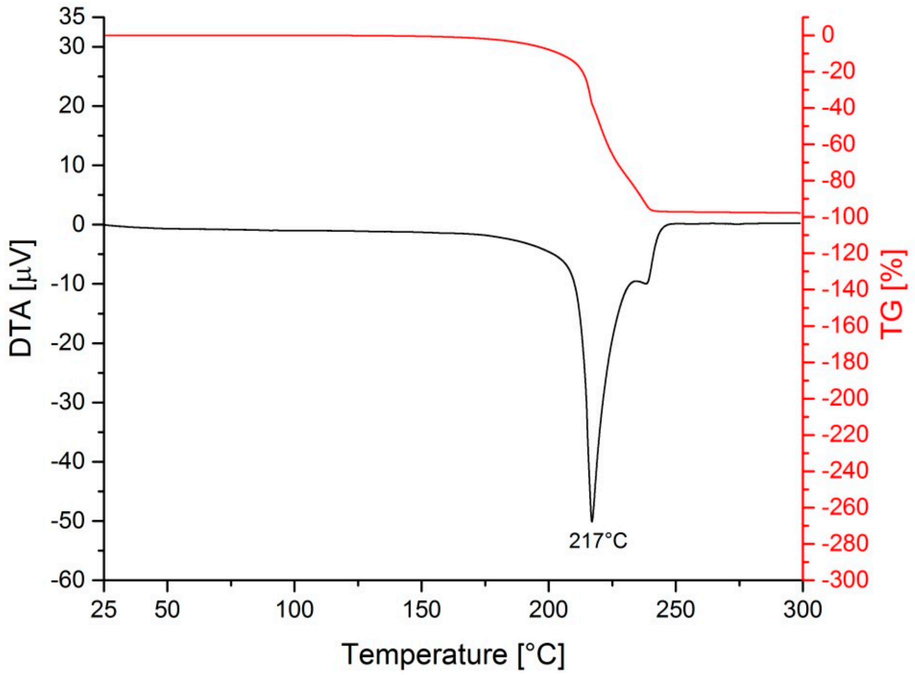

Figure S3. DTA-TG measurement of the PZA:OA (1:1) cocrystal. The first DTA signal (black) of the cocrystal at 217 °C can be assigned to the decomposition of the cocrystal as a complete mass loss is observed in the TG-analysis (red).

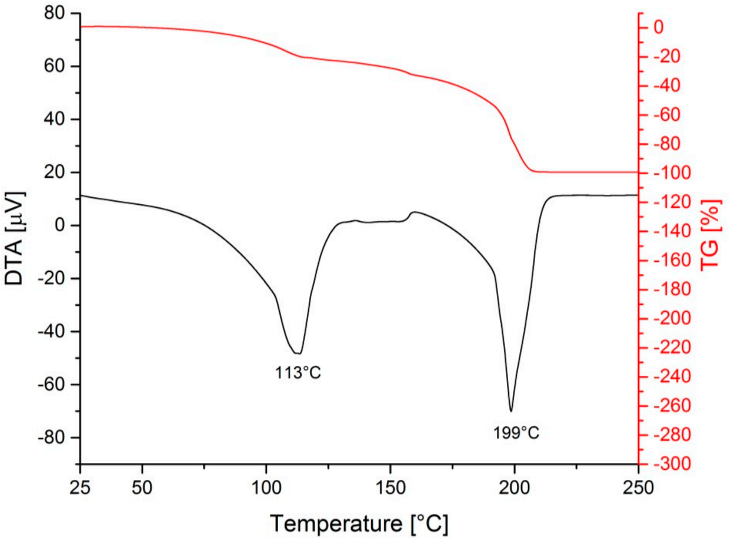

Figure S4. DTA-TG measurement of oxalic acid dihydrate. The first DTA signal (black) at 113 °C arises due to dehydration of oxalic acid dihydrate and at 199 °C a complete mass loss is in the TG-analysis (red) is observed indicating decomposition of oxalic acid.

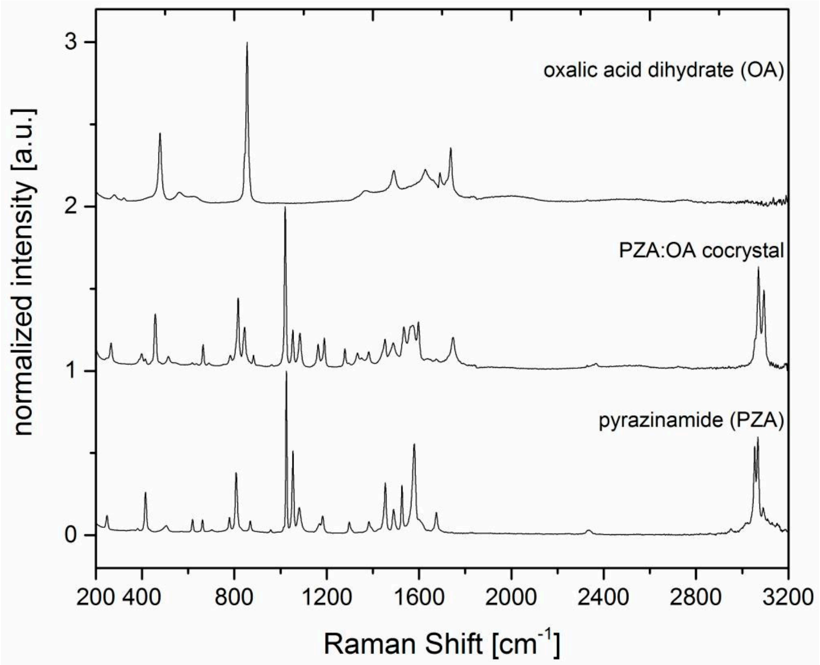

Figure S5. Raman spectra of the PZA:OA (1:1) cocrystal (center) and the reactants pyrazinamide (bottom) and oxalic acid dihydrate (top).

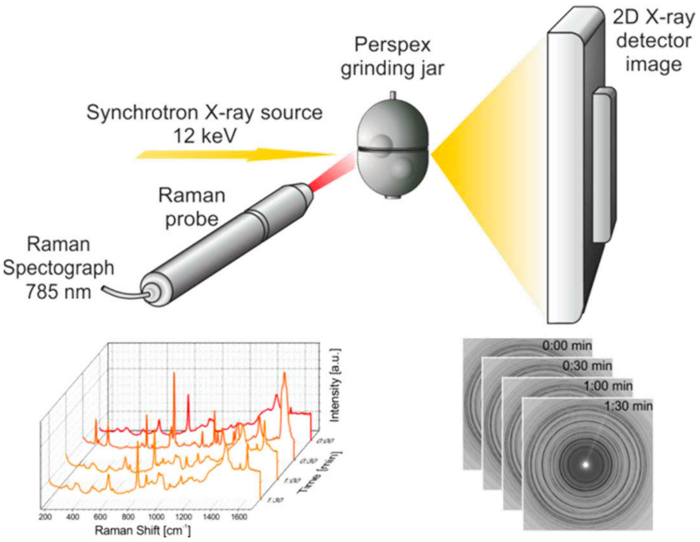

Figure S6. Scheme of the experimental in situ setup for collecting Raman spectra and X-ray diffraction patterns during the mechanochemical synthesis every 30 s [2].

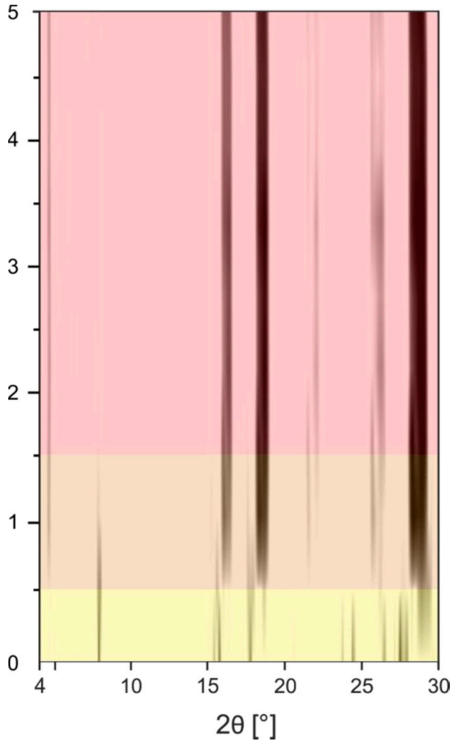

Figure S7. Time resolved reaction progress between 0 and 5 min of the synthesis process of the PZA:OA (1:1) cocrystal obtained by neat grinding of PZA with oxalic acid dihydrate followed in situ by synchrotron XRD. Yellow: reactants, orange: reactants and product, red: product.

References

1. Langford, J.I.; Louer, D. Powder Diffracton. *Rep. Prog. Phys.* **1996**, *59*, 131–234.
2. Batzdorf, L.; Fischer, F.; Wilke, M.; Wenzel, K.-J.; Emmerling, F. Direct In Situ Investigation of Milling Reactions Using Combined X-ray Diffraction and Raman Spectroscopy. *Angew. Chem. Int. Ed.* **2015,** *54*, 1799–1802.
